# Single-cell heterotrophic activity in deep-ocean prokaryotic communities quantified by BONCAT and microautoradiography

**DOI:** 10.1093/ismeco/ycag038

**Published:** 2026-02-28

**Authors:** Chie Amano, Eva Sintes, Noémie Lebon, Julia Steiger, Danilo Prijovic, Thomas Reinthaler, Ingrid Obernosterer, Kristin Bergauer, Gerhard J Herndl

**Affiliations:** Department of Functional and Evolutionary Ecology, University of Vienna, A-1030 Vienna, Austria; Department of Functional and Evolutionary Ecology, University of Vienna, A-1030 Vienna, Austria; Instituto Español de Oceanografía-CSIC, Centro Oceanográfico de Baleares; 07015 Palma de Mallorca, Spain; Department of Functional and Evolutionary Ecology, University of Vienna, A-1030 Vienna, Austria; Master de Biologie, École Normale Supérieure de Lyon, Université Claude Bernard Lyon 1, Université de Lyon, 69342 Lyon Cedex 07, France; Department of Functional and Evolutionary Ecology, University of Vienna, A-1030 Vienna, Austria; Department of Functional and Evolutionary Ecology, University of Vienna, A-1030 Vienna, Austria; Department of Functional and Evolutionary Ecology, University of Vienna, A-1030 Vienna, Austria; CNRS, Laboratoire d’Océanographie Microbienne, LOMIC, Sorbonne Université, 66650 Banyuls/mer, France; Ocean EcoSystems Biology Unit, GEOMAR - Helmholtz Centre for Ocean Research Kiel, D-24148 Kiel, Germany; Department of Functional and Evolutionary Ecology, University of Vienna, A-1030 Vienna, Austria

**Keywords:** BONCAT, HPG, single-cell analysis, prokaryotes, pelagic ocean, heterotrophic activity, ^3^H-methionine, microautoradiography

## Abstract

Prokaryotes play a central role in marine biogeochemical cycles, yet quantifying their activity requires sensitive methods, particularly in the deep ocean where their biomass and metabolic rates are low. One widely used method to determine single-cell activity of prokaryotes is bioorthogonal non-canonical amino acid tagging (BONCAT), which offers a non-radioactive approach to measure protein synthesis. However, direct comparisons between BONCAT and radioisotope-based techniques across ocean depth gradients remain limited, particularly for low-activity prokaryotic communities. To address this knowledge gap, we applied BONCAT to quantify single-cell heterotrophic activity in prokaryotic communities from surface to bathypelagic depths (1000–4000 m) in the Southern Ocean near the Kerguelen Islands. Employing picolyl azide-based copper-catalysed click chemistry, we compared BONCAT (L-homopropargylglycine [HPG] incorporation) with microautoradiography (^3^H-methionine uptake). BONCAT consistently detected active cells throughout the water column, with HPG-derived total fluorescence intensity closely correlating with both microautoradiography (R^2^ = 0.91, *P* < .001) and bulk methionine incorporation (R^2^ = 0.94, *P* < .001). This strong relationship between BONCAT and microautoradiography was maintained into the upper bathypelagic depths, where detecting single-cell activity becomes challenging. Our results demonstrate that BONCAT provides estimates of single-cell heterotrophic activity consistent with microautoradiography in deep-ocean samples, supporting its application as a non-radioactive alternative in low-activity environments.

## Introduction

The ocean is a vast reservoir of organic matter [1], and its remineralisation is largely mediated by heterotrophic prokaryotes [2]. These microbial processes are fundamental to oceanic carbon cycling and influence global biogeochemical fluxes [3]. Thus, quantifying the activity of heterotrophic prokaryotes is essential for improving estimates of oceanic carbon transformations and turnover across different carbon pools.

One of the most widely used approaches to estimate prokaryotic heterotrophic activity is based on the bulk incorporation of radiolabelled leucine [4–6], a method that provides rapid, sensitive, and specific estimates of the biomass production of the heterotrophic prokaryotic community. Bulk leucine incorporation measurements are well-suited for routine use due to the manageable incubation volumes and high reproducibility across biological replicates. The technique is particularly well suited for bathypelagic waters, where prokaryotic activity and growth rates are substantially lower than those in surface waters [7]. However, bulk measurements do not provide information at the single-cell level. When samples are processed in parallel for microautoradiography, the incorporation of radiolabelled leucine can be visualised and quantified under a microscope as silver grain halos surrounding individual cells. Moreover, microautoradiography can be combined with catalysed reporter deposition fluorescence *in situ* hybridisation (CARD-FISH) [8], allowing estimation of group-specific single-cell activity [9].

Methionine is a sulphur-containing amino acid and is essential to all organisms as it universally initiates protein synthesis. While certain taxa, such as the SAR11 clade, are methionine auxotrophs [10], many marine microorganisms are considered capable of *de novo* synthesising methionine [11]. However, since methionine biosynthesis is energetically costly [12], even non-auxotrophs readily assimilate external methionine [13], which makes it useful as a proxy for bulk heterotrophic microbial activity. Uptake of radiolabelled methionine and leucine was compared in the North Atlantic, revealing lower methionine incorporation rates than leucine [14], likely because methionine constitutes only ~2 mol % of total cellular protein amino acids in marine prokaryotes, whereas leucine typically represents ~7–9 mol % [4, 15].

More recently, bioorthogonal non-canonical amino acid tagging (BONCAT) [16], a non-radioisotope alternative for quantifying protein synthesis, expanded the application of methionine-based approaches to assess prokaryotic heterotrophic activity at the single-cell level. BONCAT relies on the incorporation of synthetic methionine analogues, such as L-homopropargylglycine (HPG) or L-azidohomoalanine (AHA), into newly synthesised proteins. These analogues contain alkyne or azide functional groups that can be fluorescently labelled via copper(I)-catalysed azide-alkyne cycloaddition (CuAAC), one of the most widely used click chemistry reactions. The copper catalyst plays a critical role in enabling this reaction, which forms the basis for BONCAT detection via fluorescence microscopy or flow cytometry [17, 18].

Compared to microautoradiography, BONCAT requires substantially shorter processing times and simpler workflows, albeit only a limited set of non-canonical amino acid substrates are available, whereas a broader range of radiolabelled compounds can be used for microautoradiography. Following initial validation on bacterial isolates as well as environmental microbial communities [19], BONCAT has been applied to a wide range of aquatic systems, from viruses [20] to eukaryotic microbes [21], in both pelagic [22, 23] and benthic [24] marine environments with most studies focusing on prokaryotic communities. Similar to microautoradiography, BONCAT provides single-cell resolution of metabolic activity via fluorescence intensity [22, 23], and can also be coupled with CARD-FISH for identification of specific taxa [22]. BONCAT generally correlates well with radiolabelled leucine incorporation [21, 22], and has also been compared with SIP-NanoSIMS [19]. However, direct comparisons between BONCAT using HPG and microautoradiography with ^35^S-methionine have shown that the two methods are not fully interchangeable. In particular, BONCAT with the filter-transfer-freeze method, in which cells retained on a polycarbonate filter are transferred onto a glass slide by freezing and subsequent removal of the filter [25], reduces background fluorescence and improves the contrast between signal and background (i.e. signal-to-background ratio [26]), but can introduce variability in cell transfer efficiency [23], which increases uncertainty in estimates of the fraction of labelled cells. This limitation is especially critical in low-biomass, low-activity samples of the deep ocean, which typically produce weak fluorescence signals.

To improve BONCAT detection sensitivity while reducing the copper-associated toxicity, picolyl azide fluorophores were introduced [27]. The picolyl group in these fluorophores coordinates Cu(I) at the reaction site, accelerating the click reaction and enabling enhanced labelling efficiency at reduced copper concentrations and this efficiency is further enhanced under chelation-assisted CuAAC [27, 28]. BONCAT using picolyl azide fluorophore has been widely applied to microbial communities in diverse environments, including soil [29, 30], hot spring sediments [31], salt marsh sediments [32, 33], and marine sediments [34]. Thus, the ability of BONCAT to quantify the heterotrophic activity at the single cell level, combined with the enhanced labelling efficiency of picolyl azide, may offer an effective approach for low-activity prokaryotic communities.

Here, we aimed at testing whether BONCAT could provide estimates of single-cell heterotrophic activity consistent with microautoradiography in deep-sea prokaryotes. We applied a BONCAT protocol using picolyl azide-based click chemistry under copper-chelating CuAAC conditions, and compared the resulting activity estimates with traditional microautoradiography using ^3^H-methionine. This comparison was conducted on natural prokaryotic communities collected from epi- to bathypelagic depths in the Southern Ocean off the Kerguelen Islands.

## Materials and methods

### Sampling and incubation

For BONCAT, we followed the protocol described by Samo et al. [23], with modifications as described below.

Seawater samples were collected during several field campaigns in the Atlantic, the Southern Ocean and the Adriatic Sea (see Table 1). To optimise the BONCAT protocol, we used aged seawater collected from 300 m depth in the tropical Atlantic during the GEOTRACES cruise (GEO), from 250 m in the North Atlantic during the MEDEA II cruise (MED), and from 0.5 m depth in the coastal Adriatic Sea off Rovinj, Croatia (ROV). These samples were collected with Niskin bottles (GEO and MED) or a bucket (ROV), transferred to acid-washed 25 L polycarbonate carboys, and stored in the dark at controlled temperature conditions for 3 months to 7 years (see Table 1). Here, aged seawater refers to seawater stored after collection without filtration, retaining the ambient microbial community, and later used directly in the incubation experiment.

**Table 1:** Seawater samples used to optimise and test BONCAT. Aged seawater was stored for an extended period at controlled temperature conditions prior to incubation with HPG. Natural prokaryotic community samples were processed immediately after collection on board. IDs are used to indicate the source of samples.

**Sample Type**	**ID**	**Location**	**Cruise** **/location**	**Sampling date**	**Sampling coordinates**	**Depth (m)**	**Inc. temp (°C)**	**Duration (h)**	**Sample condition**
Aged seawater	GEO	Atlantic Ocean	GEOTRACES (64PE321)	Jul 2010	Stn 39: 2°N, 41°W	300	4	20.4	Aged for 7 years at inc. temp
	MED	Atlantic Ocean	MEDEA II (64PE356)	Jul 2012	50–60°N, 20–40°W	250	4	20.1	Aged for 5 years at inc. temp
	ROV	Coastal Adriatic Sea	Off Rovinj	Nov 2017	45.09°N, 13.63°E	0.5	17–20	2.1	Aged for 3 months at inc. temp
Natural prokaryotic community	MOB	Southern Ocean	MOBYDICK	Feb–Mar 2018	Stn M2: 72.02°E, 50.63°S	50–500	*in situ*	5–15	Immediately processed
					Stn M3: 68.06°E, 50.68°S	50–1500	*in situ*	6–30	Immediately processed
					Stn M4: 67.21°E, 52.60°S	100–4000	*in situ*	6–30	Immediately processed

Inc. temp: incubation temperature; Duration: duration of incubation with HPG; Stn: Station

The heterotrophic activity of natural prokaryotic communities was measured at both bulk and single-cell levels in seawater samples from 50–4000 m depth in the Southern Ocean during the MOBYDICK cruise. Bulk incorporation of ^3^H-labelled methionine and leucine, single-cell microautoradiography with ^3^H-methionine, and HPG-based BONCAT were applied. These samples were collected using a conductivity-temperature-depth (CTD) rosette equipped with 12 L Niskin bottles. Contextual physico-chemical data, including microbiology from the MOBYDICK cruise, have been presented elsewhere [35]. Prokaryotic abundance was measured with flow cytometry (FACSCanto II flow cytometer, Becton Dickinson) after fixation with glutaraldehyde (final concentration 1%) and staining with SYBR Green I [36].

Triplicate aliquots, 10–40 ml for each sample, depending on the expected abundance and activity, were distributed into sterile polypropylene centrifuge tubes. One of the aliquots served as a killed control, in which 0.2 μm-filtered formaldehyde (final concentration 2%) was added prior to substrate addition. For incubations, either L-homopropargylglycine (HPG; supplied as part of the Click-iT HPG Alexa Fluor 488 Protein Synthesis Assay Kit, Thermo Fisher Scientific; final concentration 20 nM) or ^3^H-methionine (L-[methyl-^3^H]-methionine, specific activity: 80 Ci mmol^−1^, ART-0169, Biotrend; final concentration 20 nM) was added to both live samples and killed controls. Bulk leucine incorporation rates were measured in parallel as described below. The applied methionine concentration (20 nM) is higher than typical concentrations of dissolved free methionine in the oligotrophic ocean, ranging from <1 nM to ~3 nM [37, 38]. Given these low ambient concentrations, we selected 20 nM to follow our reference protocol and to be consistent with concentrations commonly used in radioisotope-labelled amino acids incorporation assays in marine microbial studies [39, 40], balancing sufficient labelling with minimal perturbation of *in situ* microbial activity. We also performed parallel incubations with AHA, applying the same concentration and incubation time; however, the fluorescence signals were substantially lower than those obtained with HPG under the conditions tested. Therefore, AHA was not used in subsequent work. This difference may be caused by several factors such as differences in analogue uptake, incorporation efficiency within the natural community, or reaction conditions, but was not investigated further in this study.

The duration of incubations varied with sampling depth: 2–7 h for epipelagic (<200 m), 12–20 h for mesopelagic (200–1000 m), and 20–30 h for bathypelagic (>1000 m) waters, based on the expected microbial activity at each depth and following the incubation times used in previous leucine-based studies [41]. Incubation times for the aged seawater samples are provided in Table 1. Samples were kept in the dark, either at the storage temperatures of the aged seawater or at *in situ* temperature ± 1°C for samples processed during the cruise. Experiments of HPG and ^3^H-methionine were conducted in parallel for each set of samples using the same volume, incubation times and temperatures. To terminate the incubations, formaldehyde (final concentration 2%) was added to the live samples. Samples were stored at 4°C for 1–18 h and subsequently filtered onto 0.2 μm white polycarbonate filters (25 mm diameter, Millipore GTTP) using nitrocellulose support filters (25 mm diameter, Millipore HAWP). Filters were rinsed twice with ~5 ml of Milli-Q water, air-dried, and stored in microcentrifuge vials at −20°C until further processing.

### BONCAT

Existing BONCAT protocols differ in click-reaction chemistry, reagent composition, and sample handling, and are often optimised for laboratory cultures or high-biomass environmental samples. In aquatic microbial ecology, BONCAT has been implemented using different CuAAC reactions, including copper-stabilizing ligands such as tris-(hydroxypropyltriazolylmethyl) amine (THPTA) [22, 42], and either standard azide dyes [22, 23] or picolyl azide-modified dyes [31, 34]. Among these, the protocol reported by Samo et al. [23] for natural marine prokaryotic communities represents the closest methodological approach for our study. Using this protocol, which employs a commercial Click-iT reagent kit (Life Technologies/Invitrogen; Thermo Fisher Scientific) on our deep-ocean samples resulted in lower fluorescence labelling than observed with microautoradiography, limiting robust detection of protein synthesis in low-activity cells. Therefore, we aimed at optimising the click-reaction chemistry and labelling conditions.

We first evaluated modifications to the baseline click-reaction following Samo et al. [23], using reagents from the Click-iT HPG Alexa Fluor Protein Synthesis Assay Kit (Thermo Fisher Scientific). Our baseline protocol included CuSO_4_ added at 2% v/v from a 100 mM stock solution (final concentration 2 mM) and an Alexa Fluor 488 azide (final concentration of 8 μM; hereafter referred to as standard azide). We tested modified reaction conditions in which either the copper concentration or the Alexa Fluor azide concentration was doubled.

To assess whether an alternative click-reaction improves detection sensitivity, we tested picolyl azide fluorophores under copper-chelating CuAAC conditions using the Click-iT Plus Alexa Fluor 488 Picolyl Azide Toolkit (Thermo Fisher Scientific). Following the manufacturer’s instructions, free copper levels in the click reaction were adjusted by mixing a CuSO_4_ stock solution with a copper protectant solution (containing CuSO_4_ and a chelator) at a 2:1 (v/v) ratio. This CuSO_4_/protectant mixture was added to the reaction buffer at 2% v/v (final CuSO_4_ concentration of 2 mM), followed by Alexa Fluor 488 picolyl azide at 5 μM. A 10 μM concentration of Alexa Fluor 488 picolyl azide was also tested. The Click-iT reagent was used here as a well-established reference. However, importantly, equivalent click-chemistry reactions can be prepared from individually sourced reagents (e.g. Vector Laboratories, formerly Click Chemistry Tools), allowing greater control over reagent composition and substantially reducing costs.

For the click reaction, a wedge of one-twelfth of a 25 mm diameter polycarbonate filter was cut with a sterile scalpel and placed into a 1.5 ml microcentrifuge tube containing 300 μL of the reaction mixture. The click reaction was performed at room temperature in the dark for 30 min, under ambient atmospheric conditions without specific oxygen exclusion. The filters were washed three times with ~20 ml of Milli-Q and dried at 37°C for 10 min.

### Cell transfer from filters

Following the click reaction, cells on the filter were transferred to microscope glass slides to reduce background fluorescence, which has been recognised as a potential technical issue [42] and is particularly pronounced on polycarbonate membrane filters [23], thereby improving the signal-to-background ratio. This step was necessary for low-activity deep-ocean samples, where background from the filter material limited single-cell detection. In high-biomass samples with stronger BONCAT signals, this transfer step may be less critical and should be evaluated depending on background levels and imaging requirements. Several transfer methods were tested: (i) the filter-transfer-freeze technique (FTF), as originally described by Hewes and Holm-Hansen [25] and later used by Samo et al. [23]; we tested both the dry-ice method and cold spray (Cytocool II, 8323, Thermo Scientific); (ii) adhesion glass slides (TOMO Adhesion Slides, Matsunami Glass Ind.) and (iii) microscope slides coated with a thin layer of adhesive material to promote cell attachment. For this third approach, we tested agarose (2% w/v), gelatine (3% w/v), and polyvinyl acetate glue, a common wood glue, diluted in Milli-Q at a ratio of 1:2 (UHU Holzleim Original, UHU GmbH).

Among these, the gelatine-based method yielded the most effective and consistent cell transfer (see Results section) and was therefore used for subsequent analyses. The detailed protocol is provided in the Supplementary Material. After filter removal, the transferred cells were mounted with a DAPI mixture consisting of 5.5 parts Citifluor (AF1, Citifluor Ltd.), 1 part of Vectashield (Vector Laboratories Inc.), and 0.5 parts phosphate-buffered saline (PBS) containing DAPI at a final concentration of 2 μg ml^−1^. To monitor potential contamination introduced during the transfer process, a blank membrane filter section was processed in parallel.

To normalise fluorescence intensity across samples, InSpeck Green image intensity fluorescence calibration beads (Thermo Fisher Scientific) were initially included as internal standards by applying them directly to the sample after the click reaction (Supplementary material). These beads allow generating a calibration curve over a wide range of fluorescence intensities, from very low (0.3% relative intensity) to the brightest signal (100% relative intensity). However, even the lowest-intensity beads were over-saturated at the exposure time chosen for our natural microbial communities, and therefore no calibration curve could be produced. Thus, bead-based normalisation was not applied in this study. Instead, a positive control filter, replicate filters of aged seawater samples with known HPG-positive cell counts and fluorescence intensity was processed alongside the samples as our positive control to ensure internal comparability throughout the analysis. All steps were conducted under dim light to minimise photobleaching of the fluorescent signal. Microscopy images were acquired within several days after mounting. To assess the effect of storage on fluorescent signals after BONCAT, we also evaluated HPG-positive cell counts and fluorescence intensity during storage at −20°C for up to two weeks.

### Microautoradiography

Microautoradiography was performed to assess single-cell methionine uptake, following the protocol described for leucine by Sintes and Herndl [9]. Similar to BONCAT, one-twelfth of each sample filter was used for microautoradiography. Emulsion preparation, exposure, development, and fixing were performed according to the manufacturer’s instructions. The nuclear emulsion (Ilford, Type K5) is light sensitive, thus all steps prior to fixing were carried out in complete darkness. Briefly, filter sections were placed onto glass slides coated with the emulsion. Subsequently, the slides were stored in a light-proof box containing silica gel desiccant and exposed at 4°C for two weeks. Following exposure, slides were developed in developer (Ilford Phenisol Developer) for 4 min, rinsed with Milli-Q, and then fixed (Ilford Hypam Fixer) for 6 min. The filter sections were peeled off, and the cells were mounted with the DAPI mixture described above. This cell transfer was required for microautoradiography, as the polycarbonate filter surface interferes with transmitted-light imaging and masks the boundaries of silver grain halos, preventing accurate detection and quantification of the halo area. The cell recovery after peeling averaged 84% (±16% SD, n = 21). Prepared slides were stored at −20°C until counting under a microscope.

### Microscopy and image analysis

BONCAT and microautoradiography slides were examined using an epifluorescence microscope (Axio Imager M2, Carl Zeiss) equipped with Zeiss filter sets for DAPI (Filter Set 49, excitation G 365, emission BP 445/50) and Alexa Fluor 488 (Filter Set 44, excitation BP 475/40, emission: BP 530/50), as well as transmitted light. Images were acquired at 1000-fold magnification using a CCD-based monochrome digital camera (AxioCam MRm, Carl Zeiss). At least 10 fields of view were recorded per filter section, where each pixel on the image corresponded to 0.01 μm^2^. The exposure time of DAPI and transmitted light images was auto-adjusted. Images of the Alexa Fluor 488 filter channel were captured with a fixed exposure time of 2000 ms.

HPG-positive cells were analysed using the Automated Cell Measuring and Enumeration tool (ACMEtool 3.0, Version 2020-07-27; Bennke et al. [26]). A minimum of 800 DAPI-stained cells was counted for each replicate (i.e. filter section). The accuracy of cell recognition by ACMEtool and cell counting software is influenced by the signal-to-background ratio. Although signal detection improved after the cell transfer step, all images were manually inspected to confirm proper cell detection. Cells with Alexa Fluor 488 and DAPI signals were considered HPG-positive. Killed controls were used to define detection thresholds and to set the baseline for Alexa Fluor 488 signal intensity, corresponding to <2% HPG-positive cells among the total DAPI-stained prokaryotes, following the threshold used in the previous study [22]. This low number observed in the killed controls also indicated negligible interference from phytoplankton autofluorescence in the Alexa Fluor detection channel. Single-cell fluorescence from the HPG was calculated as the mean grey value (MGV) multiplied by the area of the HPG-positive signal (μm^2^), divided by the incubation time with HPG (T, in hours). The MGV is defined as average pixel brightness within the detected cell area on an 8-bit scale, where 0 = black and 255 = white. Finally, the total HPG-derived fluorescence was calculated by summing the cell-specific values and expressed in MGV μm^2^ L^−1^ h^−1^.

For microautoradiography, the silver grain halo surrounding DAPI-stained cells was analysed using the AxioVision SE64 imaging software (Rel 4.9, Carl Zeiss) with a custom-made macro to measure the halo size associated with DAPI-stained cells. Similar to BONCAT, the single-cell radiolabelled methionine incorporation was estimated based on the silver grain halo area, expressed as μm^2^ cell^−1^ h^−1^. The total incorporation was calculated by summing all halo areas, expressed as μm^2^ L^−1^ h^−1^.

### Bulk methionine and leucine incorporation

Bulk substrate incorporation rates were determined using the filtration method [39]. Incubation and fixation of methionine incubations followed the procedure as described above using one killed control and two live samples per depth. For leucine incorporation, incubations were conducted with [3,4,5-^3^H]-L-leucine (specific activity: 120 Ci mmol^−1^, ART-0470, Biotrend) at a final concentration of 10 nM (≤200 m) and 5 nM (>200 m). Three live samples and two formaldehyde-killed controls (2% final conc.) were prepared per depth. Following incubations at *in situ* temperature in the dark, live samples were fixed with formaldehyde and filtered through 0.2 μm polycarbonate filters (25 mm diameter, Millipore GTTP) using nitrocellulose support filters (Millipore HAWP). The filters were overlaid and rinsed twice with 5% ice-cold trichloroacetic acid for 5 min to precipitate proteins and subsequently rinsed once with Milli-Q. The filters were dried, transferred to 20 ml scintillation vials and 8 ml of scintillation cocktail (FilterCount, Perkin Elmer) was added. Disintegrations per minute (DPM) were measured using a calibrated liquid scintillation counter (Tri-Carb 2100, Perkin Elmer) and converted to methionine or leucine incorporation rates, expressed as pmol methionine or leucine L^−1^ h^−1^.

### Analysis and presentation

All statistical analyses and data visualisation were performed in R version 4.4.2 [43]. Non-parametric tests (Wilcoxon rank-sum, Kruskal–Wallis, and Friedman rank-sum), Spearman’s rank correlation, and linear regression were used for statistical analyses. Analyses and figure generation were carried out using the following R packages: *tidyverse* [44], *ggsignif*, *rstatix*, *readxl*, *data.table*, *viridis*, *marmap*, and *ggspatial*.

## Results

### Evaluation of the cell-transfer method and sample handling

**Figure 1:**
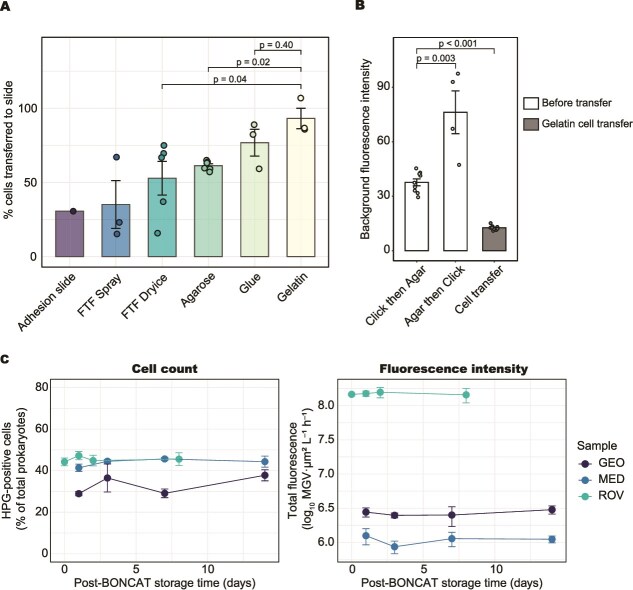
Effects of cell transfer and storage conditions on BONCAT signals and cell counts. (A) Comparison of cell-transfer methods using aged seawater samples (ROV), where filters were embedded in agarose without BONCAT. (B) Background fluorescence intensity measured after BONCAT in aged seawater samples (GEO and MED). “Agar then Click” indicates filter samples embedded in agarose before the click reaction; otherwise, the click reaction was performed first. “Cell transfer” indicates the background intensity of slides where cells were transferred using the gelatine method. (C) Relative percentages of HPG-positive cell counts and their fluorescence intensities in aged samples (ROV, MED, GEO) after BONCAT and storage at −20 °C. Statistical differences were evaluated with the Wilcoxon rank-sum test; p-values are indicated in panels A and B.

Several modifications were introduced to the baseline click chemistry protocol, resulting in improved reaction efficiency and enhanced fluorescence signal associated with HPG incorporation. To evaluate the performance of cell-transfer methods, we first quantified the cell recovery after transfer relative to the original cell abundance on membrane filters using aged seawater samples (Fig. 1A). We then assessed the background fluorescence intensity before and after cell transfer to determine the effect of transfer on signal-to-background ratios (Fig. 1B).

Cell recovery after transfer was quantified by comparing the number of cells recovered on glass slides to the original cell abundance on the membrane filters. We tested the cell transfer methods (FTF, adhesion glass slides, coating with adhesive material). We used filter sections already embedded in 0.1% low-melting-point agarose (A9414, Sigma-Aldrich), considering the potential integration with CARD-FISH to test whether cells can still be efficiently transferred to glass slides, despite the presence of the agarose layer. Our results showed that, although the FTF method and adhesion slides are clean and simple procedures, both methods resulted in low cell recovery after cell transfer (adhesion slide: 31% recovery, n = 1; FTF with cold spray: 35 ± 28%, n = 3; FTF with dry ice: 53 ± 25%, n = 5; mean ± SD, Fig. 1A). The highest recovery was achieved using gelatine (93 ± 12%, n = 3) and glue (77 ± 16%, n = 3). There was no statistically significant difference between these two methods (Wilcoxon rank sum test, *P* = .4, n = 6). Neither gelatine nor glue interfered with DAPI staining, and blank filters processed alongside the samples showed no signs of contamination from the coating solutions checked under the microscope. We selected gelatine for further use due to its easier handling than glue and resulting in a more uniform focal plane of the slide, which ensures high quality images.

We also analysed background fluorescence unrelated to the cellular signal. Compared to cells imaged directly on membrane filters, cell transfer using gelatine reduced background fluorescence by ~three-fold (Wilcoxon rank sum test, *P* < .001, n = 18) and the MGV decreased from 38 ± 6 to 13 ± 1 (n = 9), indicating that a substantial portion of the observed background originated from the membrane filter and fluorophore residues, with the apparent intensity likely enhanced by light scattering from the underlaying white polycarbonate surface (Fig. 1B). High background fluorescence can mask low levels of HPG incorporation, thus reducing background is important for detecting weak BONCAT signals. We noted that on filters already embedded in agarose prior to the click reaction, the MGV was increased to 76 ± 24 (n = 4), indicating substantial background fluorescence (Wilcoxon rank sum test, *P* = .003, n = 13, Fig. 1B). These results confirm previous recommendations to perform the click reaction prior to FISH [42]. To assess cell recovery, cells transferred to the slide and cells remaining on the filter were counted in parallel for each sample. The combined count of transferred and remaining cells, quantified on the same filters, occasionally exceeded the initial abundance by ~10%–20%, with method-dependent variability. For FTF, the large scatter in summed counts (111 ± 29% for dry ice; 112 ± 35% for spray) suggests that uneven cell transfer was the dominant source, whereas for gelatine transfer, the consistently elevated but less variable sum (115 ± 6%) was most likely due to partial cell fragmentation during filter peeling and reflects stable cell attachment to the gelatine layer. Through the cell transfer, a heterogeneous mixture of cells in terms of size, structure, and morphology has to be transferred to the glass slides. Although we did not explicitly quantify size-dependent transfer efficiency, cells remaining on the filter after gelatine transfer were predominantly low-intensity tiny signals consistent with fragments rather than intact small cells, suggesting that the selective loss of the smallest intact cells was limited. Nevertheless, when gelatine was used, more than 90% of cells remained intact, and a broad range of cell sizes and shapes (e.g. cocci, rods, elongated cells, and cells associated with particles) was preserved on the slide, indicating that fragmentation and cell loss had minimal impact on activity estimates.

Because the protocol involved multiple post-labelling handling steps, including cell transfer using gelatine, we evaluated the effect of storage duration on fluorescence intensity to determine signal stability during the processing. Multiple identical filters were prepared in parallel using aged samples and processed through the click-reaction step. The filters were mounted on four separate slides and examined after different storage durations (at T1–T4) to avoid repeated light exposure during microscopy. Images were taken for all slides under identical settings and analysed. Our results showed that storage at −20°C for up to two weeks did not affect HPG-positive cell counts or fluorescence intensity (Friedman rank sum test, *P* > .05 for both cell-count and fluorescence intensity; Fig. 1C).

### BONCAT: standard azide- vs. picolyl azide-based click chemistry

We tested several BONCAT click-reaction conditions using either standard azide-based or picolyl azide-based click chemistry performed under copper-chelating CuAAC conditions, with varying concentrations of copper and fluorophore (see Methods). The comparison was conducted using aged seawater from the GEO and natural microbial communities collected at 100 m depth from MOBYDICK (stations M2 and M4; Table 1). We compared the percentage of HPG-positive cells among total DAPI-stained cells and the total HPG-derived fluorescence signal. All comparisons were made relative to the baseline standard azide-based protocol to evaluate the differences between the protocols.

**Figure 2:**
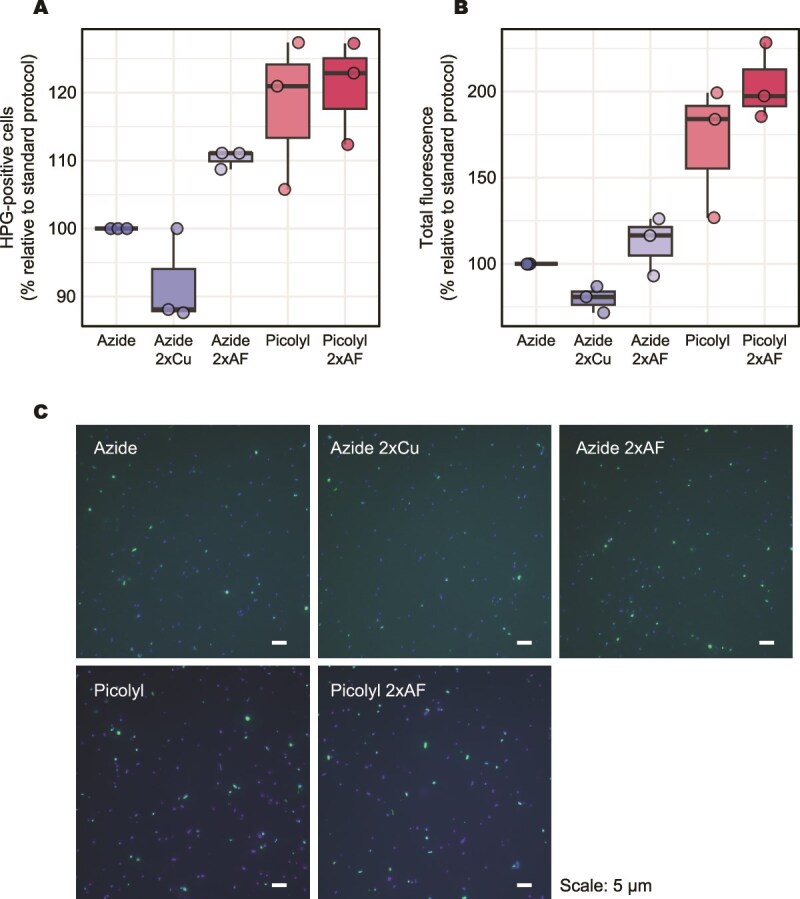
Optimisation of the click reaction using the standard azide and picolyl azide-based approaches. (A) Percentage of HPG-positive cells and (B) fluorescence intensity of HPG-positive cells expressed relative to the standard azide condition. (C) Epifluorescence micrographs of natural microbial communities incorporating HPG at 100 m depth off Kerguelen (MOB, station M4), where green fluorescence indicates BONCAT-positive (HPG-incorporating) cells and blue fluorescence corresponds to DAPI-stained cells. “Azide” refers to the standard azide-based, whereas “Picolyl” refers to picolyl azide-based approaches (see methods); “2xCu” and “2xAF” indicate doubling of the copper and fluorophore concentration, respectively.

No significant differences were observed among standard azide-based conditions (doubled copper or fluorophore concentrations) for HPG-positive cell percentages or total fluorescence intensity (Kruskal–Wallis test, *P* > .05, n = 9), whereas the use of the picolyl azide-based approach significantly increased both metrics compared to the standard reaction mix (standard azide vs. picolyl azide [5 μM and 10 μM]; Wilcoxon rank sum test, one-sided, *P* = .03 for both cell counts and intensity, n = 6). Micrographs showed lower background intensity in samples when the picolyl azide-based approach was employed compared to the standard azide (Fig. 2C), improving contrast between signal and background and facilitating detection of weak fluorescence. Varying Alexa Fluor azide concentration had little effect on the HPG-positive cell percentages but slightly increased intensity when picolyl azide was used at 10 μM (Fig. 2B).

These results suggest that using the picolyl azide-based approach enhances sensitivity and signal-to-background contrast compared to the standard azide-based approach, consistent with the previous study demonstrating the improved performance of BONCAT using picolyl azide [27]. Based on these findings, we selected picolyl azide (10 μM) as the representative picolyl condition for subsequent comparisons with the standard azide using our baseline protocol. The detailed protocol of BONCAT employing the picolyl azide-based approach is provided in the Supplementary Material.

### Bulk prokaryotic abundance and substrate incorporation off the Kerguelen Islands

**Figure 3:**
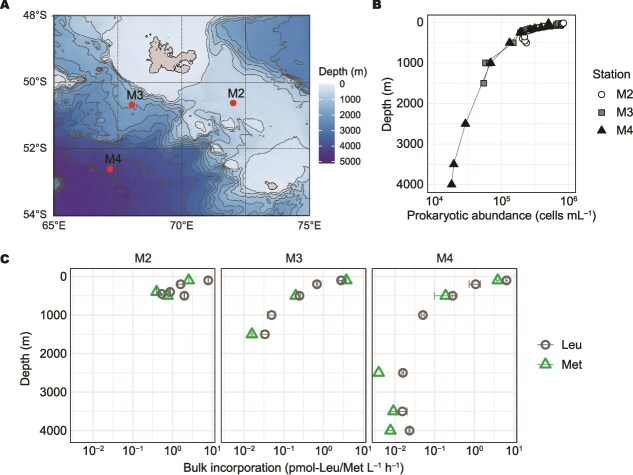
Vertical profiles of prokaryotic abundance and heterotrophic prokaryotic activity in the Southern Ocean. (A) Station map around the Kerguelen Islands. Sampling stations during the MOBYDICK cruise are indicated as red circles labelled with station names; background contours show bathymetry. (B) Depth profiles of prokaryotic abundance and (C) methionine and leucine incorporation rates at three stations. Error bars represent standard deviations.

To evaluate the applicability of BONCAT for determining protein synthesis activity in natural prokaryotic communities, we compared standard azide- and picolyl azide-based approaches on samples collected at three stations (M2, M3 and M4) with sampling depths ranging from 50 m to 4000 m during an expedition in the Southern Ocean off the Kerguelen Islands (Fig. 3A, Table 1). Station M2 was located on the plateau (bottom depth: 520 m), while M3 and M4 were located off the plateau, with bottom depths of 1700 m and 4300 m, respectively.

Prokaryotic abundance showed a typical open ocean profile, decreasing exponentially from 5–8 × 10^5^ cells ml^−1^ at 25 m depth to ~2 × 10^4^ cells ml^−1^ at ~4000 m depth (Fig. 3B). Similarly, bulk incorporation rates of radiolabelled methionine and leucine decreased with depth, reflecting the decline in prokaryotic abundance (Fig. 3C). Bulk ^3^H-methionine incorporation rates were highest in the epipelagic (3.4 ± 0.7 pmol L^−1^ h^−1^, n = 3) and decreased to 0.01 ± 0.005 pmol L^−1^ h^−1^ (n = 4) in the bathypelagic waters. Comparing the bulk methionine and leucine incorporation rates, leucine uptake was consistently higher than methionine uptake (paired Wilcoxon signed-rank test, *P* = .02, n = 11), consistent with leucine comprising a larger mol% of cellular protein than methionine, as reported by a previous study from the Atlantic Ocean [14]. Additionally, at the off-plateau stations M3 and M4, the ratio of leucine to methionine uptake increased with depth (Spearman rank correlation, ρ = 0.77, *P* = .025, n = 8). Together, these results showed a strong decline in bulk activity with depth, allowing us to test whether BONCAT can detect low-activity cells in the bathypelagic.

### Single-cell analysis of prokaryotic activity using BONCAT and microautoradiography

**Figure 4:**
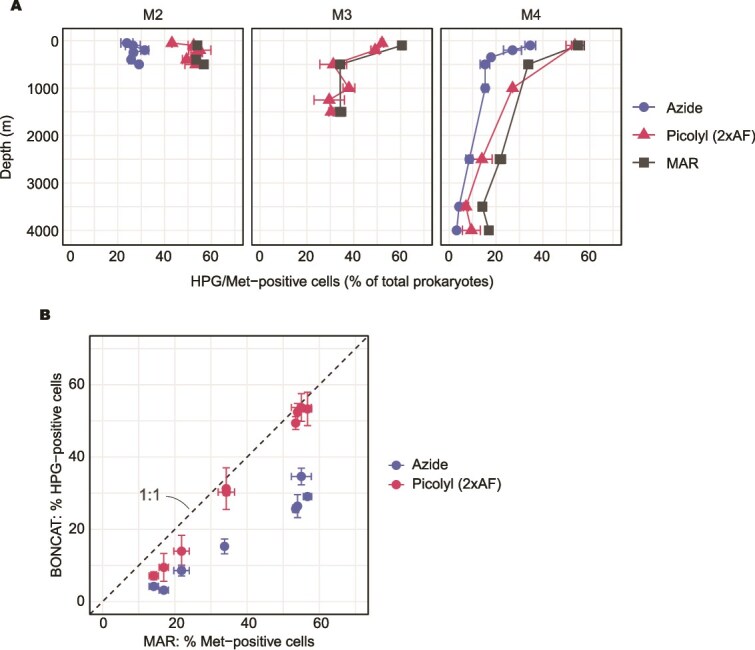
Comparison of standard azide and picolyl azide-based approaches with microautoradiography (MAR). Samples were collected from the epipelagic to bathypelagic waters during the MOBYDICK cruise and incubated with either HPG or ^3^H-methionine under identical concentrations, temperatures, and incubation times. (A) Vertical profiles of HPG or ^3^H-methionine positive cells detected by BONCAT and MAR. (B) Comparison of the percentage of HPG and ^3^H-methionine-positive cells across depths.

To assess prokaryotic activity at the single-cell level, we conducted analyses using both BONCAT and microautoradiography. For the comparison between standard azide- and picolyl azide-based approaches, samples of stations M2 and M4 were analysed. When applying standard azide, the percentage of HPG-positive cells remained relatively constant throughout the water column at station M2 (27 ± 3%, n = 6), while at station M4, it decreased from ~30% in the epipelagic layer to 8 ± 6% (n = 4) in the bathypelagic (Fig. 4A). Using the picolyl azide-based approach, the detection rates nearly doubled across depths, resulting in 52 ± 4% HPG-positive cells at station M2 (n = 7). At stations M3 and M4, the HPG-positive percentages using the standard azide-based approach were 52 ± 2% (n = 3) in the epipelagic, 32 ± 5% (n = 3) in the mesopelagic, and 18 ± 11% (n = 5) in the bathypelagic waters (Fig. 4A). These values from the picolyl azide-based approach were essentially the same as the percentages of methionine-positive cells determined via microautoradiography (Fig. 4A). At depths >2000 m, however, the relative abundance of HPG-positive cells detected by the picolyl azide-based approach tended to be lower than that detected by microautoradiography (Fig. 4A and B), indicating a difference between the two methods in the lowest activity samples (see Discussion). Additionally, the variation between technical replicates was slightly higher in BONCAT than in microautoradiography (Fig. 4B). Overall, BONCAT using the picolyl azide-based approach exhibited an ~1:1 relationship with a root mean square deviation of 3.5%, compared to 15% for the standard azide-based approach (Fig. 4B).

To detect weak fluorescence signals, a fixed long exposure time (2000 ms) was used across all samples. This improved detection in deep-water samples but caused signal saturation (MGV close to 255), in a few of the highly active cells, mostly from the epipelagic layer. However, only 0.4% of HPG-positive cells showed an MGV >225, indicating minimal signal saturation. Multiple spatially separated HPG-associated fluorescence signals within individual cells were also observed, likely reflecting heterogeneous intracellular incorporation or localised clustering of newly synthesised proteins. However, such overestimation was rare, affecting only 1 ± 3% of signals (n = 32).

### BONCAT fluorescence intensity in open ocean samples

**Figure 5:**
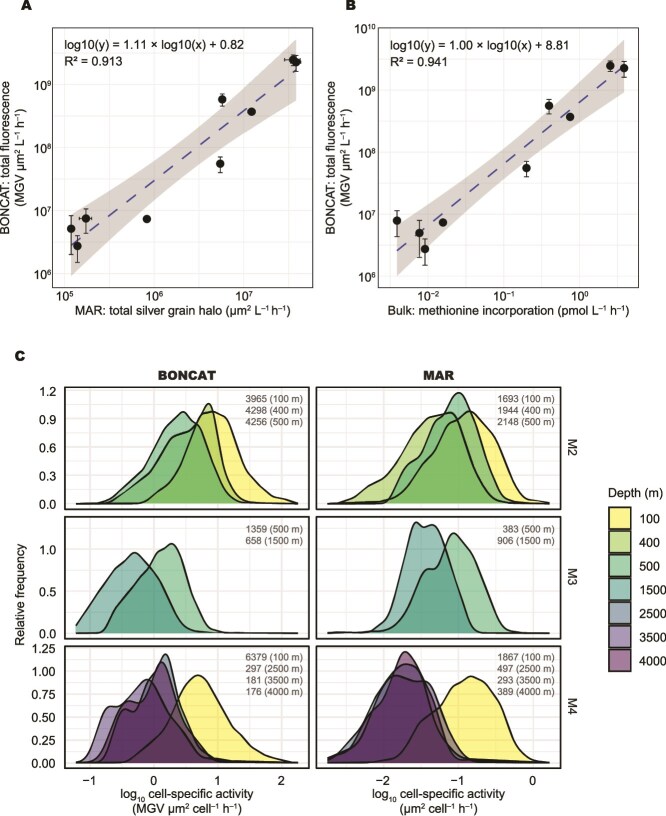
Fluorescence-based analysis of prokaryotic activity using MOBYDICK samples. (A) Total fluorescence from BONCAT versus total silver grain halo area from microautoradiography (MAR) and (B) versus bulk methionine incorporation rates. Dashed blue lines indicate regressions on log_10_ transformed data; grey shading mark 95% confidence intervals. (C) Distribution of single-cell substrate uptake activity, shown as probability density plots based on BONCAT and MAR. These plots represent the relative frequency distribution of active cells across a range of cell-specific substrate uptake rates. The number of cells analysed and the corresponding sampling depths are indicated in each panel. All BONCAT measurements were obtained using the picolyl azide-based approach (Picolyl 2xAF).

In addition to the cell count comparisons, we analysed the fluorescence signal intensity at both the community and single-cell level on the samples, where the picolyl azide-based approach was performed. These measurements were compared to the total silver grain halo area obtained by microautoradiography as well as to the bulk ^3^H-methionine incorporation rates. Total HPG-derived fluorescence intensity showed a strong positive correlation with both total silver grain halo area (R^2^ = 0.91, *P* < .001; n = 9; Fig. 5A) and bulk ^3^H-methionine incorporation (R^2^ = 0.94, *P* < .001; n = 9; Fig. 5B). Total silver grain halo area was strongly correlated with bulk ^3^H-methionine incorporation (R^2^ = 0.95, *P* < .001; n = 11). Single-cell activity decreased with depth in both HPG and methionine uptake (Fig. 5C). At station M4, cell-specific activity decreased by nearly one order of magnitude from the epipelagic to bathypelagic layer, consistent with the depth-dependent decrease observed in bulk prokaryotic activity (Fig. 3C). The relative frequency distribution of single-cell HPG fluorescence intensity closely matched that of ^3^H-methionine uptake measured by microautoradiography, with a comparable single-cell activity range of approximately two orders of magnitude.

## Discussion

Although picolyl azide has been commercially available for over a decade and is increasingly used in microbial ecology, its quantitative performance relative to radioisotope-based single-cell activity assays has not been systematically evaluated for environmental microbial communities, particularly in the deep ocean. This is mainly because direct validation requires radioisotope-based comparisons, which are technically demanding and subject to regulation. There is no leucine analogue compatible with click chemistry, restricting BONCAT to methionine-based assay. Improving the sensitivity of detecting the methionine uptake at the single-cell level is essential for quantifying microbial activity in the deep ocean, where metabolic rates are low. In this study, BONCAT using the picolyl azide-based approach significantly increased the detection of HPG-positive cells compared to the standard azide-based approach and produced results comparable to microautoradiography and bulk methionine incorporation. This demonstrates that the enhanced sensitivity of BONCAT employing picolyl azide-based click chemistry is well-suited for assessing single-cell methionine uptake in open-ocean samples. Because BONCAT requires only standard laboratory infrastructure, its accessibility also makes it a practical and widely applicable approach for routine use across a wide range of aquatic environments. Our results show that the choice of azide, which directly influences the efficiency of the copper-catalysed click reaction, significantly affects the quantification of HPG-derived signals. Picolyl azide consistently revealed a higher proportion of active heterotrophic cells across all depths compared to the standard azide (Fig. 4). Because HPG incubation and fixation conditions were identical, this difference originates from the click reaction. The copper-chelation properties of picolyl enhance the efficiency of the copper-catalysed azide–alkyne cycloaddition [27], increasing labelling efficiency and fluorescence intensity. Consequently, the proportion of HPG-positive cells was approximately doubled throughout the water column using the picolyl azide-based approach.

Because amino acid uptake is concentration-dependent [45, 46], we adjusted the substrate concentration to minimise artificially stimulating uptake while ensuring detectable labelling. Generally, elevated substrate concentrations have two opposing effects: they can suppress biosynthesis of the target compound, reducing dilution of the labelled pool, while simultaneously altering substrate uptake rates in prokaryotes [39, 46]. Therefore, the measured signal should be interpreted as potential uptake rates rather than *in situ* activity. Nevertheless, using similar nanomolar concentrations for HPG, methionine and leucine also facilitated comparisons between methods.

A key consideration of BONCAT is that incorporation of the synthetic methionine analogue HPG can affect cellular metabolism in a concentration- and time-dependent manner. Although HPG is generally used without apparent adverse effects at sufficiently low concentrations and short incubation times, effects have been reported in laboratory cultures [47, 48]. In fast-growing *Escherichia coli*, growth rate reductions were reported at 2.8 μM HPG concentrations and stronger inhibition at tens of μM, unlike ^3^H-methionine [47]. In marine *Synechococcus*, stress responses and reduced growth were reported at 25 μM HPG [48]. Given the physiological diversity of marine prokaryotes, predicting such effects in natural assemblages are difficult. In our study, we used 20 nM HPG, far below the concentrations associated with growth or stress effects in the above cited culture studies. Moreover, HPG uptake patterns were comparable to those of ^3^H-methionine in deep-ocean microbial communities (Fig. 4B), suggesting no apparent limitation and inhibition in synthetic substrate uptake. This also likely reflects the low ambient methionine concentrations in the deep ocean, where cells readily incorporate available analogues such as HPG. In oligotrophic surface waters, where dissolved free methionine concentrations are typically low, an HPG concentration of ~20 nM is therefore expected to be appropriate. However, the optimal HPG concentration is system-dependent and reflects background methionine availability. In eutrophic or methionine-rich systems (e.g. laboratory media or faecal samples), substantially higher HPG concentrations are often required to avoid dilution of the labelled pool by natural substrate.

Microautoradiography remains the standard for assessing single-cell metabolic activity, despite increasing regulatory and logistical constraints associated with radioisotope use. In this study, microautoradiography consistently detected the highest percentage of active heterotrophic cells across all stations, indicating that BONCAT, particularly with the standard azide fluorophore, may underestimate active cell populations in oligotrophic environments. In contrast, BONCAT employing the picolyl azide-based approach showed a strong correlation with microautoradiography, although detection rates were somewhat reduced in lower bathypelagic samples (>2000 m). Because only a small number of lower bathypelagic samples (n = 3) was analysed, this trend should be interpreted with caution. This apparent difference may reflect weak fluorescence signals approaching the detection threshold under low-activity conditions. In particular, incorporation of HPG is known to be less efficient than that of methionine due to the much lower catalytic efficiency of HPG by methionyl-tRNA synthetase [49]. While this difference is expected to apply throughout the water column, it becomes most apparent under low-activity conditions. Under these conditions, per-cell incorporation rates approach the detection threshold, causing HPG fluorescence signals to fall below detection earlier than those derived from radiolabelled methionine.

Converting fluorescence intensity to absolute substrate uptake rates requires careful consideration, as measurements can be influenced by factors such as signal saturation and background fluorescence. To minimise these artefacts, selecting an appropriate exposure time for microscopy is essential. Similar to the previous work [50], an additional advantage of BONCAT combined with epifluorescence microscopy is its ability to resolve subcellular fluorescence localisation within individual cells, allowing visualisation of spatial patterns of newly synthesised protein in relation to cell morphology and spatial organisation (e.g. particle-attached or dividing cells). Moreover, taxon-specific growth rates in natural communities can be estimated from the frequency of dividing cells, as demonstrated by Brüwer et al. [51]. Integrating BONCAT with such morphological or taxon-specific analyses will advance our understanding of heterotrophic activity of deep-sea microbes.

## Conclusion

This study shows that BONCAT employing the picolyl azide-based approach results in comparable estimates of metabolically active cells as microautoradiography across a broad range of oceanic depths, supporting its use for quantifying cell-specific heterotrophic activity in prokaryotic communities. The improved detection efficiency of picolyl azide resulted from its copper-chelating properties, which enhance the click-reaction efficiency and fluorescence signal strength, particularly for low-activity cells. Together, these results demonstrate that BONCAT represents a reliable and accessible method for quantifying methionine-based protein synthesis at the single-cell level in natural microbial assemblages, facilitating a deeper mechanistic understanding of microbial processes in the pelagic ocean.

## Supplementary Material

260318_clean_revisedSI_BONCATvsMAR_ISMEcom_ycag038

## Data Availability

Metadata from the MOBYDICK cruise can be found on https://doi.org/10.17600/18000403. Detailed protocols for the BONCAT used in this study are provided in the Supplementary Material. Other data can be provided upon request to the corresponding author.
