# Supplementary material for: Single-cell heterotrophic activity in deep-ocean prokaryotic communities quantified by BONCAT and microautoradiography
Source: ISME Commun. 2026 Feb 28;6(1):ycag038. doi: 10.1093/ismeco/ycag038 (PMC13064657; doi:10.1093/ismeco/ycag038)
Supplement: 260318_clean_revisedSI_BONCATvsMAR_ISMEcom_ycag038 [file 260318_clean_revisedsi_boncatvsmar_ismecom_ycag038.pdf]

Supplementary information for:

Single-cell heterotrophic activity in deep-ocean prokaryotic communities quantified by BONCAT and microautoradiography

Chie Amano*, Eva Sintes, Noémie Lebon, Julia Steiger, Danilo Prijovic, Thomas Reinthaler, Ingrid Obernosterer, Kristin Bergauer, Gerhard J. Herndl*

*Correspondence to: chie.amano@univie.ac.at, gerhard.herndl@univie.ac.at

BONCAT protocol for pelagic prokaryotes (for microscopy)

Sampling, incubation, and fixation

1. Check the cell abundance in samples to estimate the appropriate volume (Table 1).
2. Transfer seawater samples to 50 ml centrifuge conical tubes (two tubes: one for the live sample and one for the killed control). Fix the control tubes with 0.2 µm-filtered formaldehyde (final concentration 2%) and wait for 15 minutes.
3. Add substrate for both samples and killed controls, for example: 20 nM final concentration of HPG for open ocean samples. However, note that the concentrations are highly dependent on the sample; a prior check with concentration kinetics is recommended.
4. Incubate in the dark at in situ temperature. Take into account the expected activity of your sample (for example, incubation times for pelagic prokaryotes with HPG are: epipelagic: ~2-6 h, mesopelagic: ~12-20 h, bathypelagic: ~20-30h, which are slightly longer than the leucine-based assay; time-course kinetics are preferred before starting the experiment).
5. Terminate the live samples with 0.2 µm-filtered formaldehyde (final conc. 2%) and store at 4°C in the dark for 1 to a maximum of 24 h.
6. Filter the sample onto white polycarbonate filters (Millipore, GTTP, 25mm diameter), using a nitrocellulose support filter (Millipore, HAWP).
7. After filtering the sample, wash the filter twice with ~5 mL of MilliQ water.
8. Air-dry filters. Store at -20°C until processing. Filters can be kept frozen for several months.

Table 1. Example of filtration volume

Cell abundance in seawater	Volume to filter (ml)
$2x10^4$ cell ml^{-1}	~100
$5x10^5$ cell ml^{-1}	~10
$1x10^6$ cell ml^{-1}	~5

Click-chemistry

based on Samo et al. (2014), adapted for picolyl azide and copper-chelating CuAAC:

*Work under dim light conditions

1. Thaw 10xBuffer additive, and Alexa 488 picolyl azide at room temperature in the dark. Keep the 10xBuffer additive on ice once it's thawed.
2. Cut filter sections into 1/12 parts of a 25mm diameter filter and write a label on each filter section with a pencil. Additionally, cut a filter sample with a known HPG-positive cell count as a positive control, and also cut a blank new filter as a negative control.
3. Dilute the 10xBuffer additive 1:10 with MQ (e.g., 10 µL of 10xBuffer additive plus 90 µL of Milli-Q water), referred to as Buffer additive.
4. Prepare the reaction buffer (see Table 2).
5. Incubate filter sections in the reaction buffer at RT in the dark for 30 min.
6. After incubation, wash 3x in excess Milli Q water.
7. Place the filter sections on blotting paper (with the sample side up) and dry them at 37°C in a hybridisation oven for 10 min. After the click reaction, avoid exposing the sample directly to intense light; you may work in a room with dim illumination.

Table 2. Reaction buffer mixture

Stock reagent	Vol (µl)*	Vol (µl)**
Milli-Q water	154	231
10x reaction buffer	20	30
$CuSO_4$ copper protectant pre-mix	4	6
Alexa 488 picolyl azide	2	3
Buffer additive	20	30

* total 200 µl in 0.6 ml tube (~10 filter sections)

**total 300 µL in 1.5 ml tube (~15 filter sections)

FISH or CARD-FISH

If combining BONCAT with FISH or CARD-FISH, perform FISH or CARD-FISH protocol at this stage. Begin from the embedding step. Note that embedding should not be performed before the click reaction; otherwise, you will encounter high background noise.

Internal standard

* This is optional. For low-activity cells, such as pelagic prokaryotes, the bead calibration curve will far surpass their natural community signal intensity; therefore, this step can be omitted.

1. Sonicate the bead solution (0.3% intensity, 1:10 dilution with Milli-Q water) for 10 min.
2. Immediately after sonication, pipette 5 µl drops (one drop for each filter section) of the bead solution onto clean cover slips (10 drops per coverslip) and place the filter sections on top of the bead drops (with the sample side down).
3. Let the filters dry at 37°C in a hybridisation oven for 15 min in the dark.
4. After drying, carefully remove the filters from the cover slip.

Cell transfer

1. Prepare the gelatine solution in a 50 mL conical centrifuge tube (see Table 3).
2. Warm the gelatine solution to 43°C in the water bath for 15 min until it dissolves.

3. Dip a slide glass into the gelatine solution to coat it.
4. Wipe off the gelatine only from the back side of the slide. Place the gelatine-coated slide on an ice-cold aluminium plate for 1-5 min to solidify. The time depends on the laboratory's humidity. Check carefully to make sure it doesn’t dry out.
5. Place the sample filter sections on the gelatine-coated slide with the sample side facing down (max 12 pieces/slide). Add a new filter section as a control for the gelatine solution.
6. Dry the slide at RT for ~15 min (may take ~1h depending on the humidity of the lab) in the dark, until the gelatine is completely dry.
7. Mark the filter location on the side without sample filter using a permanent marker. Write down the sample ID.
8. Gently wet only the edge of the filter with an MQ-wetted cotton swab, if necessary. Carefully hold the wet edge with forceps and gently peel the filter away from the slide.
9. Mount with DAPI mix and store at -20°C until taking images with a microscope. Typically, images are captured within 24h of completing the filter section processing.

Table 3. Gelatine solution

Reagent	Volume	Final conc.
Gelatine<br>MQ	0.6 g<br>20 mL	3%

2. Buffers and Chemicals

10x Buffer additive

1. Add 2 mL of sterile ultrapure water to the bottle (Component E) and mix until completely dissolved.
2. Make 100-200 µL aliquots in sterile 0.6 mL microcentrifuge tubes.
3. Store the aliquots at ≤ –20°C. This solution is stable for up to 1 year.

Stock reagent	Volume	Final conc.
Buffer additive (Component E)<br>Sterile ultrapure water	powder<br>2 ml	10x

Alexa Fluor picolyl azide (PCA) stock (1000 µM)

Make ~50µL aliquots (see Table below) in black sterile microcentrifuge tubes and store at -20°C.

Stock reagent	Volume	Final conc.
Alexa 488 PCA (Component A)<br>DMSO	1 tube<br>105 µL	1000 µM

$CuSO_4$ copper protectant pre-mix

Stock reagent	Volume	$CuSO_4$ conc.
$CuSO_4$ (Component C)<br>Copper protectant (Component D)	200 µL<br>100 µL	100 mM

DAPI mix 2 µg/ml

Stock reagent	Volume (µl)	Final conc.
DAPI 50µg/ml	40	2 µg/ml
1xPBS	70	0.5 part
Vectashield	140	1 part
Citifluor	750	5.5 part

3. Product information

Product	Description	Company	Art.Nr.:	Size
**Alexa picolyl Azide**	Click-iT™ Plus Alexa Fluor™ 488 Picolyl Azide Toolkit	Thermo Fisher Scientific	C10641	1 kit
**Citifluor**	Glycerol/ PBS solution AF1	Citifluor Ltd. (Electron microscopy science)	17970	100 mL
**DAPI**	DAPI stain	Sigma-Aldrich	D9564	10 mg
**DMSO**	Dimethyl sulfoxide for molecular biology (DMSO)	Sigma-Aldrich	D8418-50ML	50 mL
**Gelatine**	Gelatine from bovine skin Type B, powder, BioRegent, suitable for cell culture	Sigma-Aldrich	G9391	100G
**HPG**	Click-IT™ L-Homopropargylglycine (HPG)	Thermo Fisher Scientific	C10186	5 mg
**Polycarbonate filter**	0.2 µm, 25 mm diameter	Millipore	GTTP02500	100 pcs
**Support filter**	0.45 µm, 25 mm diameter	Millipore	HAWP02500	100 pcs
**Vectashield**		Vector Laboratories, Inc.	H-1000	10 mL

Additional lab equipment, materials:

- Blotting paper, tissue paper
- Centrifuge conical tubes (50 mL)
- Ethanol for cleaning (70%)
- Fluorescence microscope (1000x magnification), Immersion oil
- Forceps, Scalpel
- Hybridization oven
- Microcentrifuge tubes (0.6 mL, 1.5 mL)
- Permanent marker, Pencil
- Petri dish
- Slide glass, Cover slip (24 x 60 mm)
- Water bath

4. Image analysis with ACMEtool3 (Bennke et al. 2016)

ACMEtool3:

https://www.mpi-bremen.de/en/automated-microscopy.html

Procedures:

*** Blue text indicates the typical image analysis settings used in our lab. These can be adjusted depending on image quality and the specifications of the microscope.**

1. Prepare TIF, 8-bit, grayscale microscopy images in a folder (usually, TIF is better quality than JPEG).
2. Choose '**1. Analyze Directory'**
3. Select the folder where the images are
4. Go to **B)** Check the photos to see if they are good.
5. Go to '**C) Image processing'**
6. Correlate '**Reference Image'** to '**Image Processing Method**'.
   Before pressing '**Add**', '**Corresponding parameters'** should be carefully checked and adjusted if necessary.
   e.g. **DAPI channel** default setting for our lab's microscopic image is:
   'Dynamic threshold', **Kernel Size: 21, Offset: 11**. If small cells are not detected with the default setting, adjust the **Kernel size to 19 and the offset to 7-9 (always an odd number)**. Also, **uncheck** '**Remove regions**'. You can also change the '**Channel Name'** and **colour.** If you are OK with the setting, press '**Add**'.
   The next channel is BONCAT-HPG (referred to as the **FITC channel;** the FITC channel is suitable for the Alexa Fluor 488 and similar dyes in our microscopy); we usually use the same settings as for the DAPI setting. These units are in pixels.
7. Start image processing and save the file. When the analysis is done, go to '**2. Load Metadata (*.IM3)'** to open the file.
8. '**Menu**' '**Expand Tree**' to see all the images.
9. Go to the '**Info/Settings**' tab, and if needed, change the '**Counting Frame'.** We usually use the default **20-pixel** offsets. When pressing the 'Show Channel Info' in the 'Info/Settings' tab, you can see the parameters applied to make the IM3 metafile (e.g., Kernel size, Offset, etc.)
10. Go back to '**FOV Browser**'—first **DAPI channel**. Check '**set**' to see the detection of the cells. Similarly, check the **FITC channel** as well. This is just to see the first detection, which usually needs to be adjusted (next step).
11. Go to '**Set Definitions**' and adjust the setting to suit your sample.
    For example, the default setting of our microscopic pictures is:
    DAPI channel: **Set Definition: Area>12 and Area<300 and SBR>1.2**
    Sub Set Definition: Nr_FITC_Signals>0
    FITC channel: **Set Definition: Area>6 and Area<300**
    Subset Definition: Area>6
    From DAPI to FITC: **Overlap Definition: Percent >0** ***(see Notes below)**
    These settings also depend on the resolution/quality of the images, which can vary across different labs. Thus, some manual inspections would help decide the setting.

Press ‘**Calculate valid cells**’. To view the detection results, go to ‘**FOV Browser**’ to see the images. Repeat this process until you are satisfied with the settings.

12. If you want to know a specific single cell information, go to ‘**FOV Browser**’ ‘**Show**’ ‘**Cell Info**’ and then choose ‘**Select**’. This information can be used to modify the ‘**Set Definition**’, whether you wish to include or exclude the target cells.
13. If you are satisfied with your setting, review all the DAPI images. If you find a poor-quality image, uncheck it to exclude it from the analysis.
14. Similarly, review all the FITC images.
15. In the ‘**FOV Browser**’, you can also check the ‘**DAPI**’ -> ‘**subset**’ to see whether you like the detection of HPG-positive DAPI.
16. After you are fine with the cell detection, go to ‘**Reports**’
17. To get cell counts per image, go to ‘**Features Sample/ FOV Report**’. The numbers displayed as output are indicated in **green text**. You can activate or deactivate by clicking the text.
18. Press ‘**Generate FOV Report**’.
19. If only a summary report is needed, press ‘**Generate Sample Report**’
20. Go to ‘**Menu**’ -> **Save** or copy the output. Paste into a spreadsheet.

Additional info (‘Cell Info’):

- Area: detected area in pixels
- Circularity: roundness (1 = perfectly round)
- Elongation: length/width
- SBR: signal to background ratio
- MGV = mean gray value (brightness of object, 0: Black, 255: White, in 8-bit images)
- MVGp90: mean of 10% brightest pixels
- MGVp10: mean of 10% darkest pixels
- MGV bg: mean gray value of the local surrounding

For single-cell intensity analysis:

- Perform ‘**1 Analyze Directory and Create Metadata**’ with the images. Use identical settings if the images are already analysed for either the Sample Report or the FOV Report (count data). For the intensity analysis, note that the **FITC** channels are placed as the first channel and **DAPI** as the second channel in the ‘**Channel List**’.
- After making the IM3 file, go to ‘**2. Load Metadata (*.IM3)’**. Apply the same settings as cell counts for ‘**Set Definition**’ except ‘**Sub Set Definition of FITC channel: Nr_DAPI_Signals>0**’, then press ‘**Calculate valid cells**’.
- Go to ‘**Report**’ -> ‘**Features Cells Report**’, choose **Sub Set** for ‘**Selection**’ and **FITC** for ‘**Channel**’, so that we can get FITC cell reports which are DAPI positive.
- Press ‘**Generate CELL Report**’.
- Save the output data.

Notes:
*An overlap threshold of Percent > 0 was used here to account for the nature of HPG-derived signals, which may occupy only a small fraction of the DAPI-defined cell area. Increasing the overlap threshold (e.g. to 20%) resulted in the loss of visually confirmed active cells in our images. Comparative tests using overlap thresholds of 0, 10, and 20% showed no difference between 0 and 10% (n = 12, natural prokaryotic communities with 20–60% HPG-positive cells), whereas 20% substantially reduced the detected active fraction. Given the low background signal and sparse cell distributions typical of open-ocean samples, false single-pixel overlaps were not observed by manual inspection. However, this overlap setting may require adjustment for samples with higher cell densities or background fluorescence.

5. References:

Bennke, C. M., G. Reintjes, M. Schattenhofer, A. Ellrott, J. Wulf, M. Zeder, and B. M. Fuchs. 2016. Modification of a high-throughput automatic microbial cell enumeration system for shipboard analyses. Appl Environ Microbiol **82:** 3289–3296. doi:10.1128/AEM.03931-15

Samo, T. J., S. Smriga, F. Malfatti, B. P. Sherwood, and F. Azam. 2014. Broad distribution and high proportion of protein synthesis active marine bacteria revealed by click chemistry at the single cell level. Front Mar Sci **1:** 1–18. doi:10.3389/fmars.2014.00048
